# Paraneoplastic leukocytoclastic vasculitis mimicking ulcus cruris as rare initial manifestation of smoldering myeloma IgG kappa

**DOI:** 10.1007/s00277-025-06366-2

**Published:** 2025-04-21

**Authors:** Christina Brummer, Kirsten Utpatel, Sebastian Geis, Sebastian Haferkamp, Andreas Brosig, Markus Herrmann, Chiara Arnreich, Joachim Hahn, Tobias Pukrop, Wolfgang Herr, Sabine Gaerditz

**Affiliations:** 1 Department of Hematology and Oncology, University Hospital of Regensburg, Regensburg, Germany; 2 Institute of Pathology, University of Regensburg, Regensburg, Germany; 3 Center of Plastic, Hand and Reconstructive Surgery, University Hospital of Regensburg, Regensburg, Germany; 4 Department of Dermatology, University Hospital of Regensburg, Regensburg, Germany; 5 Department of Transfusion Medicine, University Hospital of Regensburg, Regensburg, Germany; 6 Center of Translational Oncology, University Hospital of Regensburg, Regensburg, Germany; 7 Bavarian Cancer Research Center (BZKF), 93053 Regensburg, Germany

**Keywords:** Paraneoplastic phenomenon, Monoclonal gammopathy of cutaneous significance, Leukocytoclastic vasculitis, Skin

## Abstract

Leukocytoclastic vasculitis (LCV) has been reported as a rare paraneoplastic phenomenon associated with several hematologic disorders, including indolent lymphomas such as Waldenström macroglobulinemia. However, there are very few cases of LCV in the context of plasma cell disorders. We present the case of a 58-year-old female who developed a rapidly progressive, ulceronecrotic skin lesion on her left lower leg due to leukocytoclastic vasculitis. The lesion was initially suspected to be an ulcerative chronic wound (ulcus cruris) but represented an atypical manifestation of leukocytoclastic vasculitis as primary and only clinical sign of smoldering multiple myeloma IgG kappa. After standard induction chemoimmunotherapy with daratumumab, bortezomib, lenalidomide, and dexamethasone, the patient proceeded to high-dose chemotherapy with melphalan, followed by autologous stem cell transplantation for consolidation. Despite a bacterial skin superinfection, myeloma treatment was successfully completed without any major complications. The skin lesion healed concurrently with the reduction in paraprotein levels, and there was no need for plastic surgical intervention. LCV mimicking ulcus cruris can represent a rare and atypical initial manifestation of plasma cell neoplasia. In this case report, systemic myeloma treatment proved to be effective for inducing complete remission of advanced ulceronecrotic skin damage. This case extends the spectrum of reported monocloncal gammopathies of cutaneous significance.

## Introduction

Leukocytoclastic vasculitis is a systemic inflammatory condition primarily affecting small vessels, typically within the skin [1]. Clinically, leukocytoclastic vasculitis usually presents as palpable purpura [2]. Pathologically, it is characterized by segmental, angiocentric, fragmented neutrophils in the walls of affected blood vessels, endothelial cell damage, and fibrinoid necrosis [3]. Leukocytoclastic vasculitis is known to be associated with a variety of causes and might occur as a primary condition or secondary to an underlying systemic disease such as infections, autoimmune diseases, medications, and malignancies [4]. As paraneoplastic phenomenon, leukocytoclastic vasculitis has been previously associated with several indolent lymphomas, especially Waldenström macroglobulinemia. However, reports of leukocytoclastic vasculitis in the context of plasma cell diseases have been exceedingly rare yet (Table 1) [5].

**Table 1:** Summary of case reports on leukocytoclastic vasculitis in the context of plasma cell disorders. In total, 25 case reports about leukocytoclastic vasculitis associated with plasma cell disorders have been reported to date. Besides our case, only three other patients have been shown to display ulceronecrotic skin lesions as cutaneous manifestation of plasma cell-associated leukocytoclastic vasculitis, whereas most patients revealed diffuse palpable purpura. While most other cases found were associated with manifest MM revealing positive (slim)CRAB criteria, our patient is one of the first to report on leukocytoclastic vasculitis representing the sole and initial manifestation of smouldering myeloma further strengthening the concept of MGCS, in this case monocloncal gammopathy of cutaneous (skin) significance

Literature	Sex	Age	Initial skin lesion	Skin pattern	Skin Location	Leukocytoclastic vasculitis first manifestation of MM	MM type	High-risk genetics	CRAB criteria	Hypocomplementemia	Myeloma treatment	Skin healing under therapy
Present case	F	58	Ulcero-necrotic	local	left lower leg	yes	IgG κ	no	no	yes	Dara-VRD, HD-CTX, SCTx	yes
McMillen, 1986 [6]	F	58	purpura	diffuse	Trunk, limbs	Yes	IgA κ	N/A	Yes (A)	no	Vincristine, melphalan, cyclophosphamide, prednisolone	yes
Means, 1987 [7]	M	64	N/A	N/A	N/A	Yes	IgA κ	N/A	N/A	N/A	N/A	N/A
Benet, 1988 [8]	M	60	N/A	N/A	N/A	Yes	IgG κ	N/A	N/A	N/A	N/A	N/A
Benet, 1988 [8]	M	70	N/A	N/A	N/A	No	IgA λ	N/A	N/A	N/A	N/A	N/A
Kois, 1991 [9]	F	75	purpura	diffuse	ankles	Yes	IgG λ	N/A	yes	N/A	N/A	N/A
Nousari, 2000 [10]	F	76	purpura	diffuse	Lower legs, trunk	yes	IgG κ	N/A	Yes (A)	no	N/A	N/A
Bayer-Garner, 2003 [5]	M	58	purpura	N/A	trunk	N/A	IgA λ	N/A	Yes	N/A	N/A	N/A
Bayer-Garner, 2003 [5]	F	67	purpura	N/A	Chest	N/A	N/A	N/A	Yes	N/A	N/A	N/A
Bayer-Garner, 2003 [5]	F	42	purpura	N/A	Leg	N/A	IgA κ	N/A	Yes	N/A	N/A	N/A
Bayer-Garner, 2003 [5]	M	67	purpura	N/A	trunk	N/A	IgG κ	N/A	Yes	N/A	N/A	N/A
Bayer-Garner, 2003 [5]	M	57	purpura	N/A	Leg	N/A	IgG κ	N/A	Yes	N/A	N/A	N/A
Bayer-Garner, 2003 [5]	M	73	purpura	N/A	Arm	N/A	IgG κ	N/A	Yes	N/A	N/A	N/A
Bayer-Garner, 2003 [5]	M	66	Purpura	N/A	Arm	N/A	IgG λ	N/A	Yes	N/A	N/A	N/A
Bayer-Garner, 2003 [5]	F	41	purpura	N/A	leg	N/A	N/A	N/A	Yes	N/A	N/A	N/A
Sánchez, 2004 [11]	F	73	Ulcero-necrotic	diffuse	lower legs	yes	IgG κ	N/A	Yes (B)	no	melphalan, dexamethasone	No
Witzens, 2004 [12]	M	62	purpura	diffuse	Lower legs	no	IgG κ	N/A	Yes (B, A)	N/A	VID, HD-CTX, SCTX	Therapy-induced
Min, 2006 [13]	M	55	purpura	diffuse	Trunk, back, limbs, face	no	IgA κ	N/A	Yes (B, A)	N/A	VAD Melphalan, Prednisolone, Vincristine, BCNU, Cyclo	Therapy-induced
Carlesimo, 2011 [14]	F	71	purpura	diffuse	fingers	no	IgA λ	N/A	Yes (R)	N/A	melphalan, dexa	yes
Peterlin, 2011 [15]	F	58	purpura	diffuse	Lower limbs, chest, trunk, feet	yes	IgA λ	N/A	Yes (B, A)	N/A	VD	yes
Cağırgan, 2012 [16]	F	35	Ulcero-necrotic	diffuse	face, legs	yes	IgG κ	N/A	Yes (C, R, A, B)	N/A	VAD, HD-CTX, SCTx	yes
Abouzaid, 2013 [17]	F	48	purpura	diffuse	Arms, legs	yes	IgA λ	N/A	Yes (A, R)	no	VAD	yes
Oka, 2018[18]	F	85	purpura	diffuse	lower legs, chest, trunk	yes	IgG κ	no	Yes (R, A)	yes	VRD	yes
Umemura, 2018 [19]	F	78	Ulcero-necrotic	diffuse	Lower legs	yes	IgA λ		no	N/A	prednisolone	No
De Abrew, 2020 [20]	F	76	Ulcero-necrotic	local	sacral	no	IgA λ	no	Yes (B)	N/A	VCD	No

## Case description

**Fig. 1:**
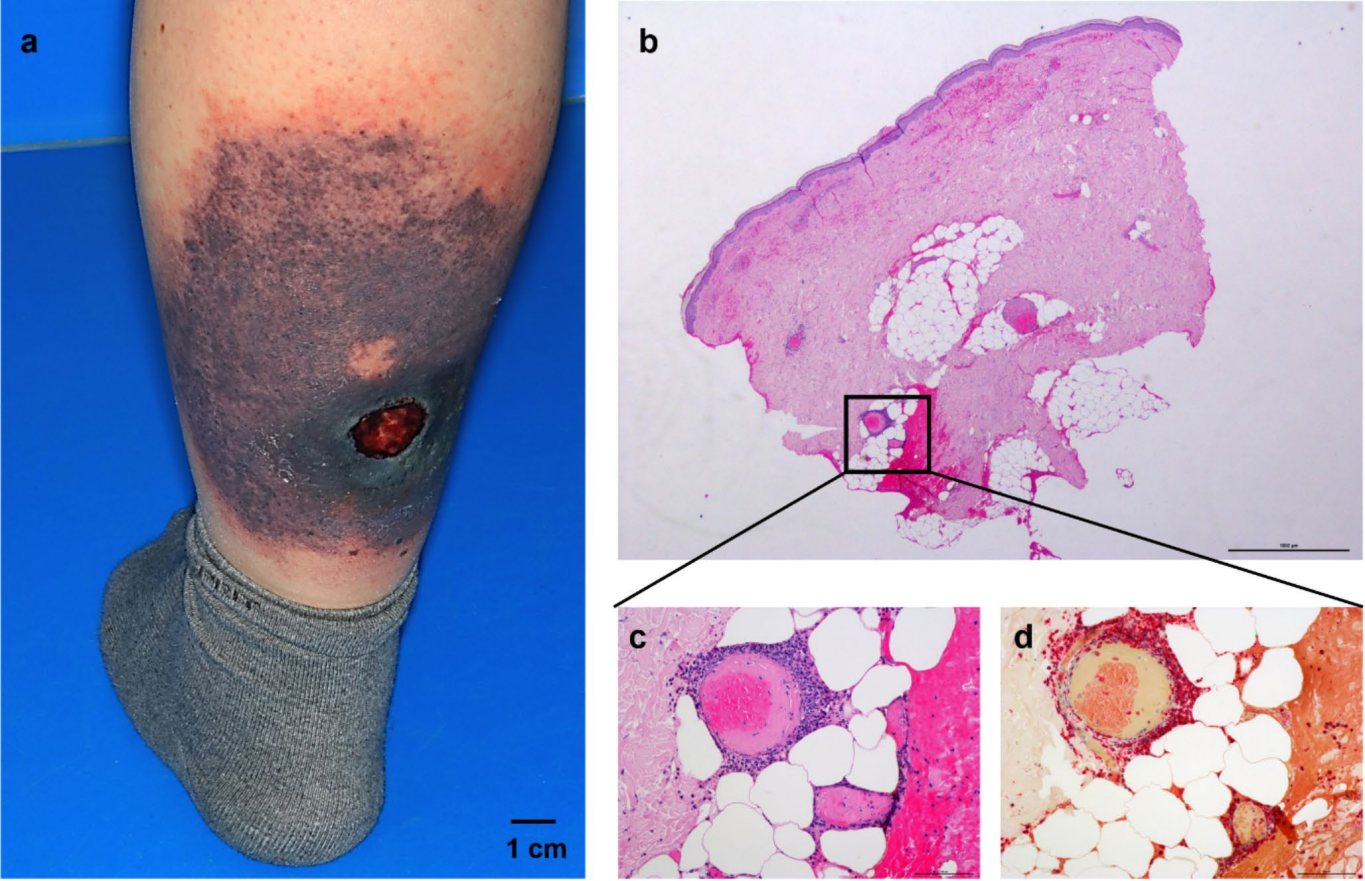
Macroscopic and microscopic imaging of the skin lesion at initial patient presentation: (**a**) Clinical examination revealed livid confluent macules (10 × 15 cm) with a 2 × 2 cm ulceronecrotic lesion on the left lower leg. (**b**, **c**) Hematoxylin and eosin stain (HE) staining of the skin biopsy showing extravasation of erythrocytes. Blood vessel walls display perivascular infiltration of neutrophils, often with nuclear debris, which is typical for leukocytoclastic vasculitis (b: Magnification: 20x, c: Magnification: 200x). (**d**) In the chloroacetate staining, the numerous granulocytes appear red (Magnification: 200x)

A 58-year-old female without any chronic illnesses or past medical issues was admitted to our dermatology department in December 2023 due to a rapidly progressing skin lesion on her left lower leg. The patient reported the onset of spontaneous hematoma on her left calf two weeks prior which progressively developed into an ulcer. The patient had no history of trauma, chronic vein insufficiency, diabetes, arteriosclerosis or any underlying rheumatic or infectious pre-condition. Clinical examination revealed livid confluent macules (10 × 10 cm) on the lower left leg with a 2 × 2 cm ulceronecrotic lesion (Fig. 1a). Sonography showed superficial vein thrombosis of the left great saphenous vein, but arterial flow profiles were physiological excluding acute limb ischemia. Standard laboratory tests indicated no abnormities displaying normal blood counts, kidney and liver function parameters, electrolytes, lactate dehydrogenase and basic inflammatory markers such as c reactive protein (CRP) and procalcitonin (PCT).

**Fig. 2:**
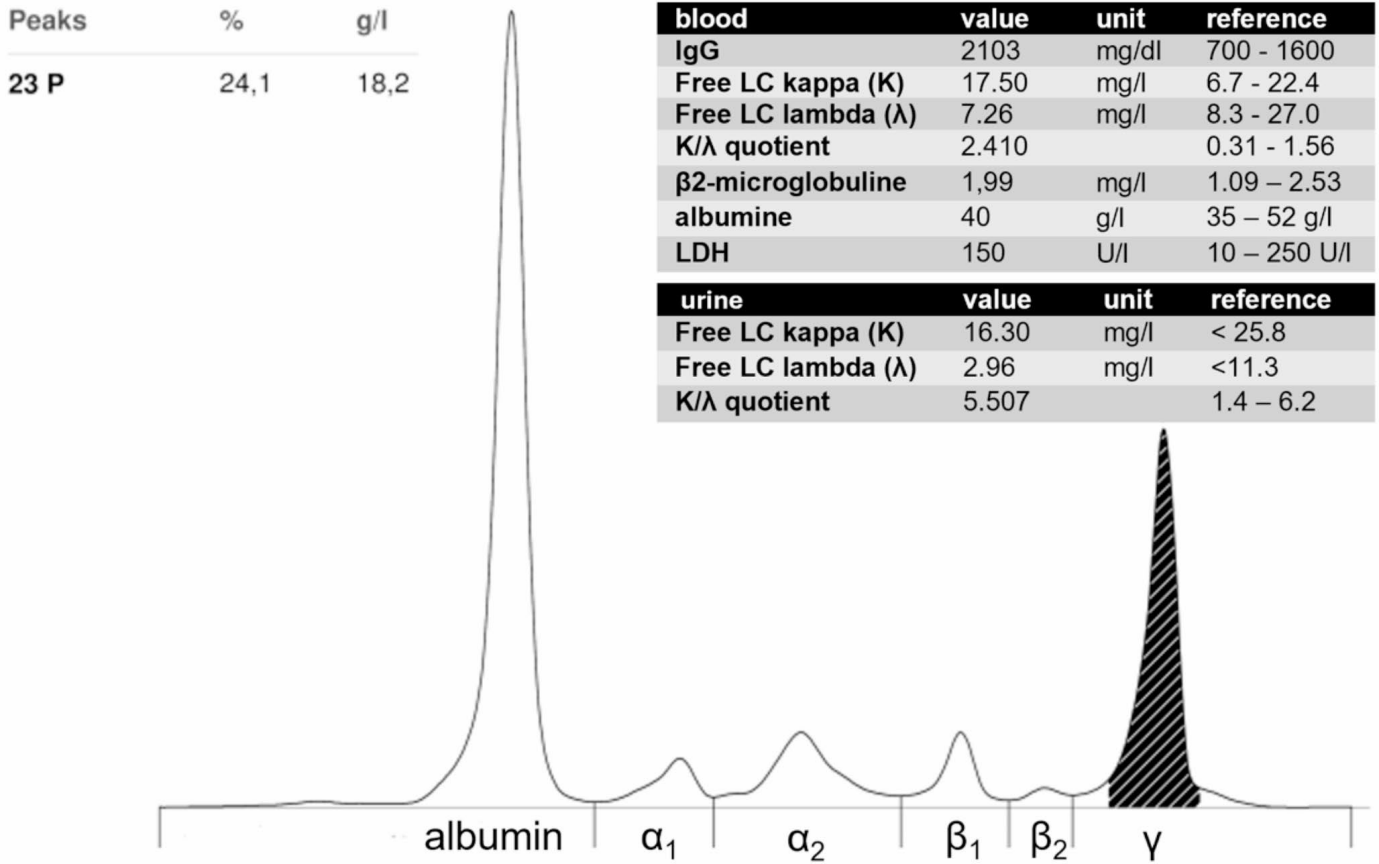
Monoclonal gammopathy IgG kappa as underlying condition of leukocytoclastic vasculitis: Protein electrophoreses revealed a significant M-gradient in the γ fraction (peak 23P: 24,1%, 18.2 g/l). Immunofixation confirmed monoclonal gammopathy IgG kappa with light chain (LC) kappa restriction. In line, the serum kappa/lambda (Κ/λ) quotient was increased. Albumin, β2-microglobuline and lactate dehydrogenase (LDH) were normal

For further diagnostics a punch biopsy was performed, which revealed perivascular infiltration of neutrophils with nuclear debris in the blood vessel walls, typical for leukocytoclastic vasculitis (Fig. 1b-d). Staining for amyloid and immunocomplexes was negative. While rheumatic screening tests including anti-neutrophil cytoplasmatic antibodies (ANCAs), anti-nuclear antibodies (ANAs), anti-double-stranded DNA (anti-ds DNA), anti-cyclic citrullinated peptide (anti-CCP) and rheumatic factor, cryoglobulins and hepatitis serology were unremarkable, an M-gradient was detected in the protein electrophoresis (Fig. 2). C3 and C4 were lowered, indicating complement consumption. Additional paraprotein analysis revealed a significant increase in monoclonal immunoglobulin G (IgG) with kappa (κ) light chain (LC) restriction. In line, the kappa/lambda quotient (κ/λ) was increased. Immunofixation confirmed monoclonal gammopathy IgG κ.

**Fig. 3:**
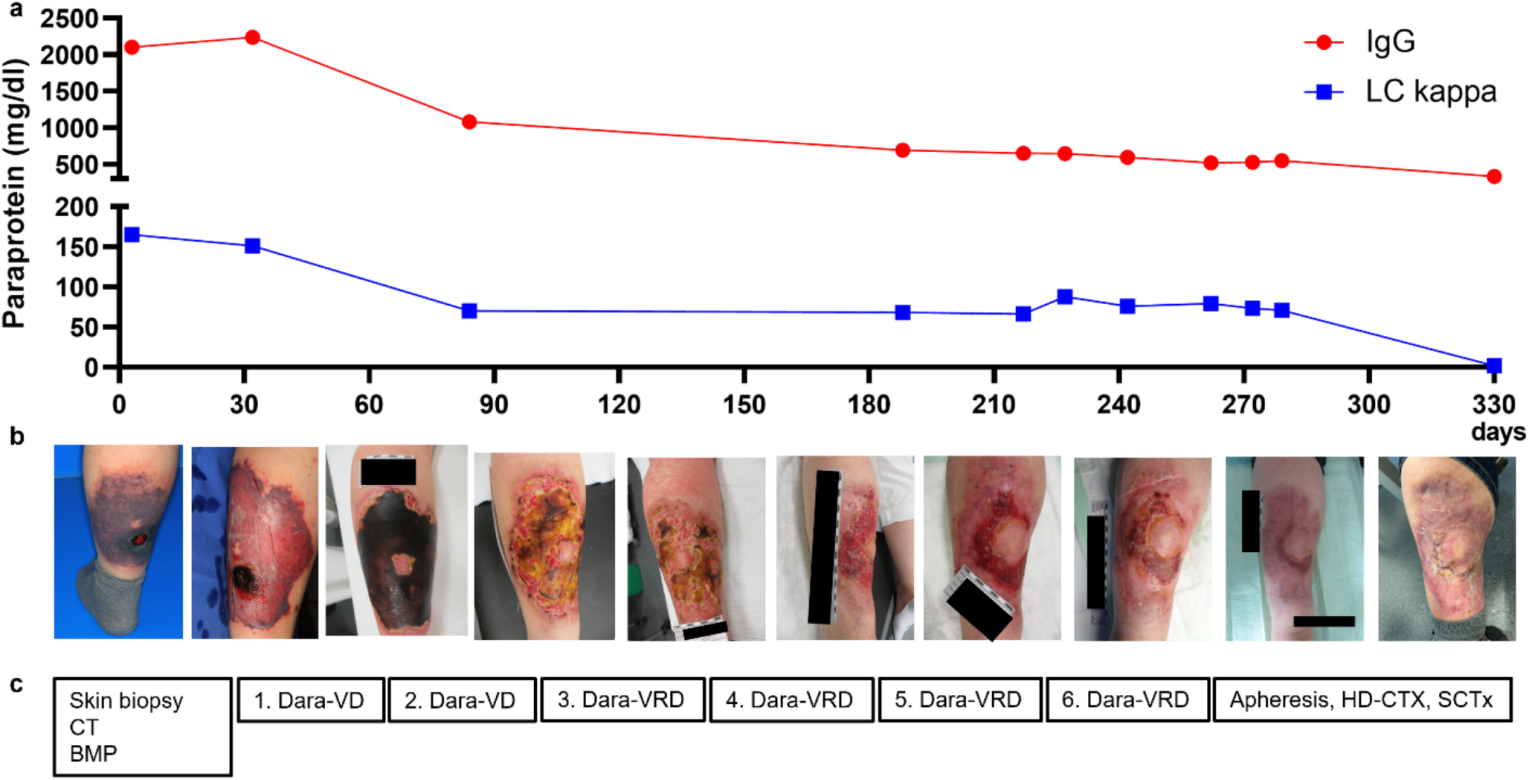
Time course of serum paraprotein levels (**a**) in relation to skin healing (**b**) and systemic myeloma treatment **(**c**)** After diagnosis of MM-associated leukocytoclastic vasculitis, the patient was treated with six cycles of Dara-V(R)D. After completion of standard induction chemotherapy, apheresis, high-dose chemotherapy with melphalan and consecutive autologous stem cell transplantation was performed. To protect the patient‘s privacy, name and date of birth were blacked out in the pictures. The scale shown indicates 7 cm. Figure 3a was created with GraphPad Prism (V8, GraphPad Software, La Jolla, CA)

Suspected of having multiple myeloma as the underlying cause of the leukocytoclastic vasculitis, the patient was admitted to the hematology department for further diagnostics and treatment. Serum albumin (40 g/l), β2-microglobuline (1,99 mg/l) and lactate dehydrogenase (LDH) values (150 U/l) were normal. Bone marrow biopsy revealed 20% plasma cell infiltration with LC κ restriction. Cytogenetic analysis showed a normal karyotype (46, XX), molecular genetic analysis detected a 1q gain (1q21) and translocation t(11,14). Computed tomography of the bones detected no signs of osteolysis. Thus, the patient fulfilled no slimCRAB criteria (Fig. 2) and based on the detection of > 10% bone marrow infiltration of clonal plasma cells the diagnosis of smoldering myeloma IgG kappa (R-ISS I, low risk according to the IMWG 2-20-20 risk stratification model) was established. The leukocytoclastic vasculitis, as a sole clinical manifestation of paraproteinemia, indicated monoclonal gammopathy of cutaneous (skin) significance (MGSC). After case discussion at the local tumor board, we decided to admit the patient to chemoimmunotherapy since literature review of similar cases often reported MGSC responding to systemic myeloma therapy (Table 1). The patient was initiated on standard myeloma induction chemotherapy, consisting of the anti-CD38 antibody daratumumab (dara), bortezomib (velcade), lenalidomide (revlimid), and dexamethasone (dexa) analogous to the PERSEUS study [21]. In the meantime, the skin lesion progressed to a necrotic plaque, and the patient exhibited significantly elevated inflammatory markers. A skin swap confirmed bacterial superinfection of the skin lesion with *staphylococcus aureus* and *klebsiella oxytoca*. Antibiotic therapy with piperacillin/tazobactam was administered for 10 days. Wound care was performed with dry dressings every two days, in close collaboration with the dermatology and plastic surgery teams. Due to a running cost coverage request to the patient’s health insurance company, the first two cycles of induction chemotherapy were administered without immunomodulatory drugs (IMiDs). After just two courses of Dara-Vd, a significant decrease in serum paraprotein was observed (Fig. 3a). Additionally, the necrosis on the left lower leg had demarcated and could be surgically debrided by the plastic surgeons. A follow-up skin swab revealed clearance of *Klebsiella oxytoca*, but persistent colonization with *Staphylococcus aureus*. However, as the patient showed no systemic signs of infection (normal temperature and inflammatory markers) anymore, antibiotic therapy was discontinued. After successful cost coverage request, the induction chemotherapy could be escalated to Dara-VRd including lenalidomide upon cycle three. Besides an intermittent drug-induced rash, which limited the maximum tolerated dose of lenalidomide to 5 mg/d (d1-21), no adverse effects or complications occurred, and the induction chemotherapy could be completed in an outpatient setting for the whole period. The addition of the IMiD lenalidomide to the induction chemotherapy scheme led to further improvement of the chronic wound on the left lower leg which eventually resolved under conservative wound care and systemic myeloma treatment without any need of plastic surgery intervention (Fig. 3b-c).

After four well-tolerated cycles Dara-V(R)D, the patient was evaluated for stem cell apheresis. Clinical examination of the skin lesion showed significant improvement of the local findings. However, microbiological analysis still indicated mixed flora bacterial colonization (s*taphylococcus aureus*,* enterobacter cloacae)*. To minimize the risk of bacterial contamination of the collected stem cells, we decided to postpone apheresis and to continue with two more cycles of Dara-VRd. After six cycles of induction chemotherapy, the skin lesion had improved further. According to our local standard for stem cell mobilization, the patient was stimulated with granulocyte colony stimulating factor (G-CSF, 5 µg/ kg body weight, 2x/d) for four days for steady state mobilization to prepare for apheresis. On day 4 of stimulation, stem cells were first detected in the peripheral blood and collected. Apheresis continued daily for 4 days to obtain the required number of CD34 + cells. Microbiological analysis of the stem cell apheresis product showed no signs of contamination. Subsequently, the patient underwent consolidating high-dose chemotherapy with melphalan (100 mg/m², each d-3 and d-2) and consecutive autologous stem cell transplantation of 3.11 × 10^6^ CD34 + cells (d0). During chemotherapy-induced neutropenia, the patient showed again rising inflammatory markers (CRP_max_ 354 mg/l). Clinical examination indicated no clear evidence of infectious source. Blood culture analyses revealed bacteraemia with *methicillin-sensitive staphylococcus aureus*; probably derived from the chronical wound on the leg. The patient was treated with antibiotics intravenously, initially with meropenem and vancomycin, which was rotated to cefazoline (due to prior allergic reaction to penicillin) after receiving the microbiological evidence of *staphylococcus aureus.* Further diagnostics (CT, skin examination, eye examination, echocardiography) showed no evidence for septic embolies. During the bacteriemia, the patient was cardiopulmonary stable without any need for oxygen supply or catecholamines all the time. Subsequently, inflammatory parameters decreased and neutrophile as well as thrombocyte take was observed on day + 13 after transplantation. A follow-up skin swap after completion of antibiotic therapy indicated no evidence of bacterial contamination anymore. The patient could be discharged on day + 26 after stem cell transplantation.

**Fig. 4:**
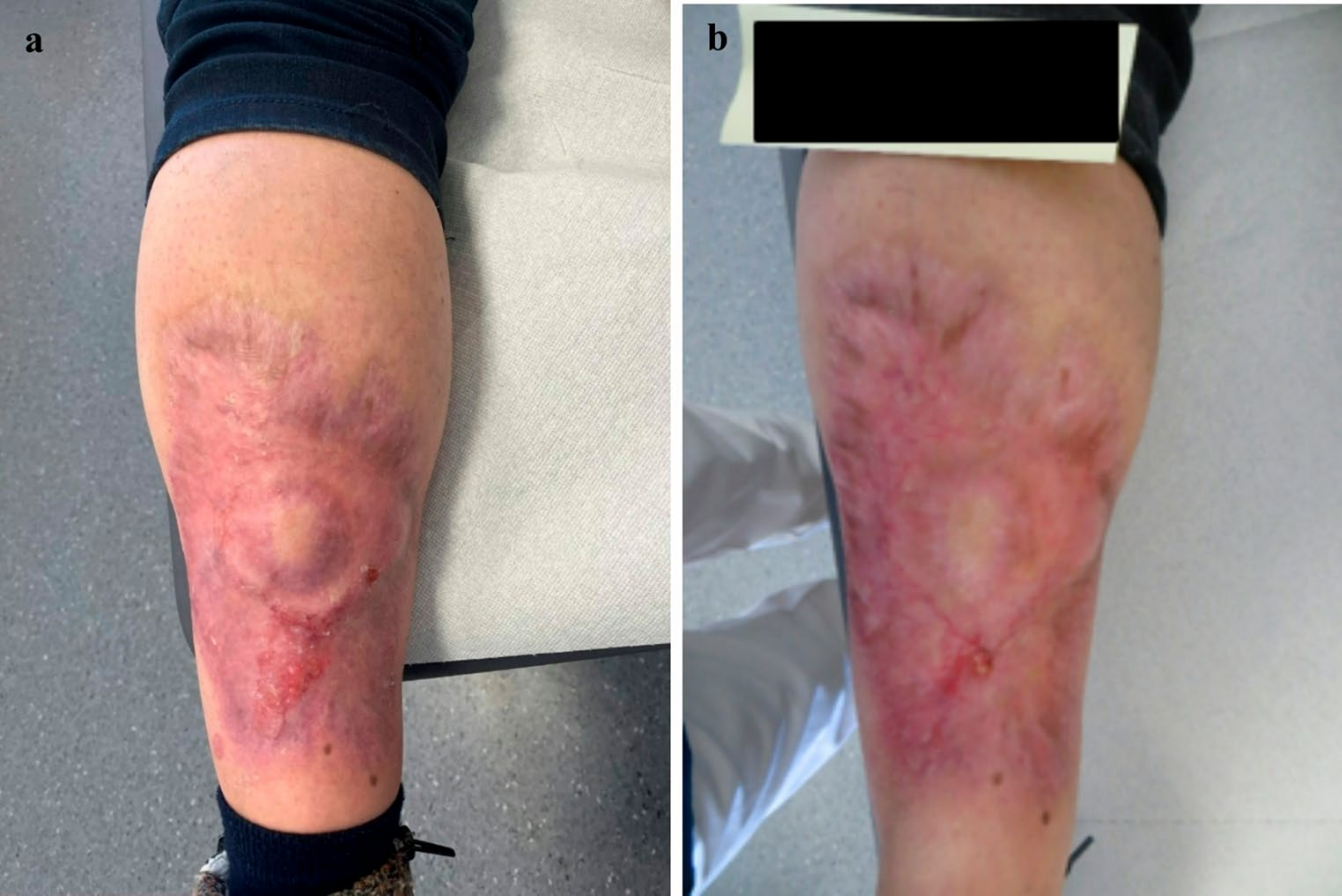
Macroscopic imaging of the skin lesion after completion of systemic myeloma therapy After completion of standard induction chemotherapy followed by consolidating high-dose chemotherapy with consecutive autologous stem cell transplantation, the skin lesion on the left lower leg was nearly completely resolved without the need of any plastic surgery intervention. The pictures were taken 4 months (**a**) and 5 months (**b**) after stem cell transplantation. To protect the patient‘s privacy, name and date of birth were blacked out in the pictures

At the follow-up control 12 weeks after stem cell transplantation, the patient showed completely regenerated blood counts, improving cardiovascular fitness and overall well-being. Paraprotein analysis indicated serological complete response while a follow-up bone marrow punction was not performed. The skin lesion on the left lower leg was almost completely resolved (Fig. 4a). For maintenance therapy, lenalidomide was initiated at the previously maximum tolerated dose (5 mg/d). Subsequently, the patient was regularly seen in the oncological day clinic for follow-up visits, last in March 2025. To date, the patient has maintained persistent serological CR and demonstrated good tolerance to maintenance therapy with lenalidomide (5 mg/day), without any adverse effects. Meanwhile, the skin lesion has further improved (Fig. 4b). Regular follow-up visits are scheduled.

## Discussion

Given the rarity of paraneoplastic leukocytoclastic vasculitis in the context of plasma cell disorders, case reports remain the primary source of evidence. To date, 24 other cases of leukocytoclastic vasculitis have been reported (Table 1), all associated with either IgA or IgG myeloma [5–15, 17–20, 22]. The cutaneous lesions of leukocytoclastic vasculitis typically presented as diffuse palpable purpura, predominantly affecting the limbs or the trunk [5–15, 17–20, 22]. Thereby, leukocytoclastic vasculitis mostly displayed the initial manifestation of therapy-indicating multiple myeloma [6, 14, 15, 17, 18, 22]. Fewer cases have been reported where leukocytoclastic vasculitis developed during myeloma treatment, with some being attributed to therapy-related factors such as IMiDs or proteasome inhibitors [12, 13].

Here, we present an atypical case of leukocytoclastic vasculitis mimicking ulcus cruris as sole and initial manifestation of smoldering myeloma IgG kappa, which was successfully treated with induction chemoimmunotherapy (Dara-VRd) followed by high-dose chemotherapy and autologous stem cell transplantation. To the best of our knowledge, we are the first to report on leukocytoclastic vasculitis in the context of smoldering myeloma IgG, where the paraneoplastic skin lesion served as the primary indicator of monoclonal gammopathy and defined treatment indication, despite the absence of any slimCRAB criteria. We identified only one similar case, which described leukocytoclastic vasculitis associated with monoclonal gammopathy of undetermined significance (MGUS) type IgA λ. However, in this case, leukocytoclastic vasculitis was treated only with prednisolone and recurrently relapsed upon corticosteroid withdrawal [19]. In our patient, therapeutic management of the underlying smoldering myeloma was successful for addressing the vasculitis and the skin lesion resolved completely without the need for plastic surgery upon decrease of paraprotein.

The effectiveness of chemotherapy in patients with leukocytoclastic vasculitis secondary to manifest multiple myeloma (MM) has been described in several reports before [6, 14, 15, 17, 18, 22]. However, to date, no standardized treatment protocol has been established, and the therapeutic approach for patients with MGUS associated with paraneoplastic skin lesions remains unclear (Table 1). The presented case highlights that individual adaptation of standard chemoimmunotherapy protocols is crucial for the management of these complex cases. In our patient, we have determined the indication for myeloma treatment, even though none of the slimCRAB criteria were met. Furthermore, we postponed apheresis upon cycle six of induction chemotherapy to allow further wound consolidation and treatment of bacterial superinfection to minimize the risk of bacterial stem cell contamination. The concept of “monoclonal gammopathy of clinical significance” (MGCS) defines a subgroup of patients with paraproteinemia which present with associated organ damage or systemic syndromes but do not meet the diagnosis criteria for plasma cell or lymphoproliferative disorders [23–25]. The main organ systems affected by MGCS are the kidney (MGRS), the nervous system (MGNS), the eyes (MGOS) and the skin (MGSS) [23–25]. While renal MGUS-associated manifestations have been newly defined as MGRS in the 5th edition of the World Health Organization (WHO) classification of plasma cell neoplasms and paraprotein-producing disorders in 2022, other MGCS are still subject of current research [23–25]. The presented case further extends the spectrum of reported monoclonal gammopathies of cutaneous (skin) significance and strengthens the benefit of systemic myeloma therapy in these complex cases [26, 27]. Notably, only recently the results of the “AQUILA” study have been published demonstrating that high-risk SMM patients benefit from daratumumab monotherapy compared to active monitoring [28]. However, since patients with MGCS were not included in this study, it remains unclear whether CD38 monotherapy would have yielded similar results in the case presented here as by combined immunochemotherapy. Additionally, our patient was classified as having low-risk SMM, and comparable studies for this subgroup are still lacking.

While most reports about plasma cell disorder associated leukocytoclastic vasculitis describe diffuse palpable purpura as leading cutaneous manifestation, local ulceronecrotic lesions, as in the presented case, are an exceedingly rare and atypical presentation of leukocytoclastic vasculitis: Besides our case, only three other patients have been reported displaying ulcerations as cutaneous manifestation of paraproteinemia-associated leukocytoclastic vasculitis. Sanchez and colleagues presented the case of a 73 old female patient who presented with diffuse ulceronecrotic skin lesions on the lower legs, hypercalcemia, bone lesions and impaired kidney function. Subsequently, the patient was diagnosed with multiple myeloma IgG κ and treated [11]. Only recently, De Abrew et al. reported on a 76 years old female who presented with an ulceronecrotic sacral skin lesion mimicking multiple myeloma IgA λ [20]. Cağırgan et al. presented the case of a 35 years old female with diffuse ulcero-necrotic skin lesions in the face and legs which was the initial manifestation of multiple myeloma IgG κ [22].

The pathogenesis of leukocytoclastic vasculitis in multiple myeloma is the subject of current research. Multiple influencing factors have been discussed, including the deposition of immune complexes, altered cytokine production, endothelial dysfunction, and activation of the complement system. These processes trigger an inflammatory cascade, leading to neutrophil infiltration and damage to the vessel walls, which are hallmarks of leukocytoclastic vasculitis. In line with previous reports [18], we found significantly reduced serum levels of complement C3 and C4, indicating complement consumption as possible trigger for the paraneoplastic leukocytoclastic vasculitis in this case.

## Conclusion

Leukocytoclastic vasculitis represents an extremely rare paraneoplastic manifestation of multiple myeloma, typically showing up as diffuse palpable purpura. Here we report on an atypical case where leukocytoclastic vasculitis presented as localized ulceration on the left lower leg, which mimicked ulcus cruris but represented the initial manifestation of smoldering myeloma IgG κ. Despite representing at a stage of disease which usually does not yet warrant treatment, systemic myeloma therapy led to complete resolution of the large, ulceronecrotic and superinfected skin lesion, without any need for plastic surgery. This case extends the spectrum of reported monoclonal gammopathies of cutaneous significance and demonstrates that timely recognition, case-by-case decision upon systemic treatment indication and collaborative efforts between pathologists, oncologists, dermatologists and plastic surgeons are crucial for the effective therapeutic management of these complex cases.

## Declarations

### Data statement

The raw data supporting the conclusion of this article will be made available by the authors, without undue reservation.
